# Brain Prehabilitation for Oncologic Surgery

**DOI:** 10.1007/s11912-022-01312-1

**Published:** 2022-07-28

**Authors:** Neil Daksla, Victoria Nguyen, Zhaosheng Jin, Sergio D. Bergese

**Affiliations:** 1 Department of Anesthesiology, Stony Brook University Health Science Center, Stony Brook, NY 11794-8480 USA; 2 Department of Neurosurgery, Stony Brook University Health Science Center, Stony Brook, NY 11794-8480 USA

**Keywords:** Postoperative delirium, Postoperative cognitive dysfunction, Frailty, Cancer, Surgical prehabilitation, Cognitive prehabilitation

## Abstract

**Purpose of Review:**

This review aims to summarize the current research on postoperative cognitive complications, such as delirium and cognitive dysfunction. This includes discussion on preoperative preventive strategies, such as physical and nutritional prehabilitation as well as up-to-date information on neuroprehabilitation.

**Recent Findings:**

Current recommendations for prevention of postoperative delirium have focused on multicomponent interventions. The optimal composition of surgical prehabilitation programs targeting exercise and nutrition has not yet been established. The Neurobics Trial shows that cognitive prehabilitation improves cognitive reserve and may be a useful addition to multimodal surgical prehabilitation.

**Summary:**

Perioperative management of oncologic patients is often associated with a myriad of challenges, such as the management of tumor-related pathologies, adverse events from neoadjuvant therapy, and chronic metabolic and immunological changes associated with malignancy. In addition, oncologic patients are at increased risk of developing frailty, which adversely affects postoperative recovery and further cancer treatment. As a result, oncologic patients are at considerable risk of developing postoperative cognitive complications, such as delirium and cognitive dysfunction. In this review, we discuss the effect of prehabilitation on postoperative cognitive outcomes.

## Introduction

Postoperative cognitive complications include delirium and cognitive dysfunction. The development of such events is associated with considerably worse clinical outcomes, longer hospital stay, and higher morbidity and mortality, as well as higher healthcare costs [1–4]. In higher risk patients undergoing high-risk surgery, the incidence could be as high as 50% [5]. Even with further research into the pathophysiology of postoperative delirium, treatment options remain limited. However, up to 40% of cases may be preventable [6]. As a result, current recommendations have focused on risk stratification and reduction measures. Frailty is not routinely assessed but has been found to be significantly associated with postoperative delirium [7•, 8]. It is estimated that over half of patients undergoing oncologic surgery may be classified as either frail or prefrail [9]. This is likely multifactorial as a result of cancer-related pathologies and patient behavioral changes. Surgical prehabilitation aims to address these modifiable risk factors during the presurgical period in order to reduce postoperative complications [10]. Cognitive prehabilitation has not been as extensively studied but shows promise as a preoperative strategy to reduce the risk of postoperative delirium. In this review, we will discuss the issues of postoperative cognitive complications as well as conventional and cognitive prehabilitation.

## Postoperative Cognitive Complications

Clinically significant postoperative neurocognitive complications affecting older patients include postoperative delirium (POD) and postoperative cognitive dysfunction (POCD). Delirium, defined as an acute change in attention and cognition, is the most common postoperative complication in older patients, affecting up to half of older patients following surgery [11]. The incidence of POD ranges from 11 to 46% after cardiac surgery and 13 to 50% after major noncardiac surgery [11–13]. Delirium has been found to have long-term implications, including prolonged cognitive and functional impairment [11].

The most widely used tool to identify delirium is the Confusion Assessment Method (CAM) demonstrating a sensitivity of 94% and specificity of 89% in high-quality studies including over 1000 patients [12]. The CAM-ICU consists of four criteria: (I) acute fluctuating mentation, (II) inattention, (III) disorganized thinking, and (IV) altered level of consciousness. Patients that meet both criteria I and II, as well as either III or IV, are diagnosed with delirium and labeled as CAM-ICU positive [14].

The development of delirium is likely multifactorial in older patients and involves the interaction between multiple predisposing and precipitating risk factors. Predisposing factors include increasing age, frailty, disability, alcohol or drug abuse, multiple comorbidities, living in institution, and severity of illness [12, 13]. Additional predisposing factors can be classified by system. Neuropsychological factors include cognitive impairment, dementia, prior stroke, depression, history of delirium, and limited cognitive reserve [13, 15]. Cardiovascular factors include history of hypertension, heart failure, and ischemic heart disease [12, 13]. Respiratory factors include chronic obstructive pulmonary disease, obstructive sleep apnea, and current smoking [12, 13]. Gastrointestinal factors include history of diabetes mellitus, malnutrition, low albumin, and high body mass index [12, 13]. Furthermore, the risk of POD is also influenced by the type of surgery and medication exposure. Commonly identified precipitating factors include intraoperative factors (surgical complexity, surgical duration, surgical approach, cardiopulmonary bypass, transfusion, depth of sedation/burst suppression) and postoperative factors (infection, pain, hypoxemia, mechanical ventilation) [13]. Exposure to certain medications, such as benzodiazepines, ketamine, opioids such as morphine or meperidine, anticholinergics such as diphenhydramine or scopolamine, sleep aids such as zolpidem, and histamine receptor antagonists, has also been associated with a risk of developing POD [13]. However, current evidence precludes recommendations on specific anesthetic agents or doses, regional/neuraxial blockade as the primary anesthetic, or the administration of prophylactic medications to reduce the risk of POD [13]. Finally, the Successful Aging after Elective Surgery (SAGES) study was a long-term prospective cohort study that provided the groundwork for future studies by examining novel risk factors for delirium including genetic and plasma biomarkers (e.g., CRP), neuroimaging markers, life-course factors (e.g., early childhood factors such as family income), and cognitive reserve markers (e.g., occupational complexity) in elderly patients without preexisting dementia undergoing elective major surgery [11, 16, 17].

Given the multifactorial nature of POD, a single pathophysiological mechanism has been difficult to establish. Instead, several interacting sets of biological factors may contribute to the disruption of neuronal networks in the brain, leading to acute cognitive dysfunction [12]. One possible mechanism involves alterations in neurotransmitters, with the most commonly implicated being a relative cholinergic deficiency and/or dopamine excess. Neurotransmission can be affected directly by a variety of factors including drugs, hypercortisolism, electrolyte disturbances, hypoxia, or impaired glucose oxidation [12]. Another possible mechanism involves inflammatory cytokines leading to neuroinflammation and neuronal injury. Other hypothesized mechanisms include physiologic stressors, metabolic derangements, electrolyte disorders, and genetic factors [12].

Postoperative cognitive dysfunction (POCD) also occurs in a substantial number of older surgical patients and shares similar risk factors to POD [18]. POCD is defined by a decline in cognition postoperatively compared with preoperative function as measured by neuropsychological tests [18, 19]. The cause of POCD remains unknown. However, factors such as increasing age, baseline cognitive impairment, and fewer years of education are consistently associated with POCD [19]. The incidence of POCD ranges from 20 to 50% of older patients 3 months after cardiac surgery and 5 to 55% of those undergoing noncardiac surgeries [18, 20].

Although POD and POCD share many of the same risk factors, they are distinct neurocognitive disorders. Daiello et al. [18] used data from the SAGES study and demonstrated that POD increased the risk of POCD at 1 month postoperatively; however, there was no association between POD and POCD at 2 and 6 months after major noncardiac surgery. In fact, POCD was more common among patients without delirium. Similarly, Franck et al. [21] performed secondary analysis of a previous study of elderly patients undergoing noncardiac surgery and could not find evidence of independent association up to 3 months.

The development of postoperative cognitive complications such as POD or POCD is associated with adverse outcomes to both the individual and healthcare system. Delirium is associated with persistent impairments in brain function including cognitive decline and increased risk of dementia, decline in physical functioning, increased mortality, and death [11–13, 16, 18]. The development of delirium is also linked to longer hospital length of stay, increased risk of institutionalization, increased risk of readmission, and significant healthcare costs ranging from $38 billion to $152 billion each year in the USA [4, 11, 13]. POCD has been associated with delay in returning to work, premature retirement, and increased mortality [18].

## Frailty and Cognitive Complications

Frailty describes a clinical state characterized by multisystem decline associated with loss of physiological reserve. This can be thought of as a quantitative summation of measurable clinical parameters (including comorbidity and functional impairments) [22], or as a clinical syndrome distinct from functional impairment or disability alone [23]. When frail persons are exposed to a stressor such as chemotherapy or cancer surgery, they are at increased risk for developing disability or dying [9, 24, 25]. In the general population, frailty affects approximately 10% of people aged 65 and over and 25–50% of those aged 85 and over [7•, 9].

While there are numerous assessments that can be utilized to identify frailty in older patients, the FRAIL Scale serves as a brief questionnaire that can be used by nonmedically trained personnel [9, 14, 24]. The FRAIL Scale examines five items: fatigue, resistance, ambulation, illnesses, and loss of weight. A score of 0 is categorized as fit, 1–2 as prefrail, and ≥ 3 as frail [9, 14, 24]. The Edmonton frailty scale is an example of a more involved assessment undertaken through interview and physical tasks. There are 11 items with variable weighting that adds up to a total score of 17, with those scoring more than 5 being described in various stages of frailty [26].

In a prospective cohort study, Mahanna-Gabrielli et al. [14] examined the relationship between preoperative frailty and postoperative cognitive complications after major noncardiac surgery [14]. They found that patients who tested frail or prefrail with the FRAIL Scale had 2.7 times the odds of developing POD (97.5% confidence interval 1.0 to 7.3) when compared to patients who were fit. In addition, there was no association between frailty and POCD in older noncardiac patients. Similarly, Susano et al. [24] used the FRAIL Scale to show that older patients undergoing elective surgery had a high prevalence of frailty, and frailty along with cognitive impairment was associated with POD. Gracie et al. [7•] subsequently conducted a meta-analysis of 9 studies and a total of 794 patients. The authors reported that patients with frailty were at significantly higher risk of developing POD (odds ratio 2.14, 95% confidence interval 1.43 to 3.19). As previously mentioned, POD is associated with increased risk of dementia, decline in physical functioning, and increased mortality and death. Thus, the identification of and treatment of frailty is an essential component of preoperative management in older patients undergoing elective surgery.

## Cancer and Frailty

As the population ages, the number of older adults with cancer increases. It has been well-established that older adults have an increased prevalence of frailty, which puts them at increased risk for adverse surgical outcomes and increased risk for developing disability or dying when exposed to stressors such as chemotherapy. Handforth et al. [9] evaluated observational studies that showed more than half of older cancer patients have prefrailty or frailty. The pathophysiology linking cancer and frailty is likely to be multifaceted, including metabolic and immune dysfunction, cancer therapy-related polypharmacy, and direct effect of the tumor as well as functional changes [27, 28]. Patients with frailty are at increased risk of chemotherapy intolerance, postoperative complications, and mortality [9]. For example, a systematic review and meta-analysis by Boakye et al. [29] showed that frailty was a strong prognostic factor of survival in colorectal carcinoma patients. Another systematic review and meta-analysis by Ding et al. [30] found that preoperative frailty was associated with an increased risk of postoperative complications and mortality in patients with digestive system tumors. Furthermore, Kwon et al. [31] reported the importance of utilizing a frailty screening tool focused on respiratory and swallowing functions in older patients with head and neck cancer as this patient population had an increased risk of developing functional disabilities associated with respiration and swallowing that significantly affected early morbidity and mortality. As a result, the identification of older patients with prefrailty or frailty who may benefit from preoperative optimization is vital to guide treatment in vulnerable patients such as the geriatric oncology patient population.

## Preoperative Strategies to Prevent Delirium

Although not currently approved by the Food and Drug Administration (FDA) for the treatment of delirium, haloperidol and second-generation antipsychotics remain popular choices in intensive care patients [32]. Early clinical trials suggested that they were effective for the management of delirium symptoms [33–35]. However, a meta-analysis by Neufeld et al. [36] showed that the use of antipsychotics did not improve outcomes with no association in change in delirium duration, severity, or hospital length of stay. This finding is also supported by a recent systematic review by Nikooie et al. [37]. Moreover, although they found no difference in mortality, potentially harmful cardiac side effects tended to occur more frequently.

With the lack of effective pharmacologic options, the focus should be on risk stratification and reduction. In fact, up to 40% of delirium cases may be preventable [6]. It makes sense that with the number of different predisposing risk factors, any intervention should involve multiple components. The success of this strategy could be seen with the Hospital Elder Life Program (HELP), which targeted six different risk factors, including cognitive impairment, sleep deprivation, immobility, visual impairment, hearing impairment, and dehydration, in hospitalized older patients. Interventions included reorientation, participation in cognitively stimulating activities, promotion of sleep hygiene, early mobilization, visual and hearing adaptations if needed, and maintenance of hydration. The intervention group was found to have reduced incidence and duration of delirium [6]. Similar protocols were later adapted for the perioperative period resulting in reduced incidence and length of stay [38, 39]. In addition, a systematic review by Siddiqi et al. [40] also found strong evidence that multicomponent interventions can prevent delirium in both medical and surgical settings.

As part of the American Society of Anesthesiologists (ASA) Perioperative Brain Health Initiative, an expert panel recently came up with six consensus-based recommendations for reducing the incidence of POD. These include education and training programs, preoperative baseline cognitive screening with a validated test and assessment of additional risk factors, delirium screening with a validated screening tool before discharge from the recovery room and twice daily until day 5 or discharge from the hospital, multicomponent nonpharmacologic interventions, multimodal pain control, and avoidance of antipsychotics and benzodiazepines for first-line treatment of delirium. These were comparable to the strongest recommendations from the American Society for Enhanced Recovery and Perioperative Quality Initiative [13, 41].

Similarly, potential preoperative strategies for preventing POD should target multiple components. Comprehensive geriatrics assessment (CGA) is a multidisciplinary approach for evaluating the medical, psychosocial, and functional needs of older patients in order to develop an individualized plan for preoperative optimization, treatment, and postoperative recovery [42]. Partridge et al. [43] conducted a randomized trial of elderly patients undergoing vascular surgery, in which the intervention group received preoperative CGA by a multidisciplinary team. This facilitated identification of risk factors, medication review, discussion with the patient and family, communication with the ward team, multicomponent interventions, and social work referral. The intervention group had shorter hospital length of stay and lower incidence of complications, including POD. A systematic review by Shields et al. [44] found similar results when looking at four different randomized controlled trials of CGA in elderly patients with hip fractures. However, a systematic review by Eamer et al. [42] concluded that more studies are still required to determine whether CGA can improve outcomes in surgical patients other than those presenting with hip fractures. Furthermore, CGA may make little or no difference in delirium rates.

Surgical prehabilitation aims to improve baseline function and physiologic reserve before surgery in order to facilitate recovery and reduce postoperative complications. For oncology patients, this occurs during the period between cancer diagnosis and surgical treatment. It often encompasses exercise, nutritional, and psychological interventions [10]. A randomized clinical trial by Minnella et al. [45] showed that exercise and nutrition prehabilitation improved functional capacity in patients before and after esophagogastric cancer surgery compared with the control group. Systematic reviews have found a reduction in postoperative complications, decreased length of hospital stay, and improved quality of life [46–48]. However, the optimal composition of surgical prehabilitation programs has not yet been established and requires more research. The conclusions from a systematic review of prehabilitation programs in abdominal cancer surgery by Hijazi et al. [49] were limited after finding heterogeneity in the number of components, duration of prehabilitation, type and intensity of exercise, and outcome measures. Nevertheless, a systematic review by Thomas et al. [50] found that despite this heterogeneity, the studies with high therapeutic validity showed improved postoperative outcomes.

Preoperative nutritional state is thought to be an important determinant for the risk of postoperative cognitive complications [51]. Retrospective studies have reported that when controlled for confounding factors such as age, comorbidities, and baseline cognitive function, nutrition remains an independent risk factor for POD [52, 53]. Similarly, preoperative physical activity level was also found to be strongly associated with POD risk [54]. Janssen et al. [55•] conducted a before-and-after study in elderly patients undergoing elective abdominal surgery for colorectal carcinoma or abdominal aortic aneurysm. After being assessed for risk factors, patients in the intervention group received home-based personalized exercise programs from a physiotherapist, dietary instructions from a dietician, and CGA from a geriatrician, resulting in a significant reduction in delirium incidence. Further supporting evidence for the utilization of nutrition and exercise-related interventions in the prevention of POD could be derived from studies which investigated the use of exercise and nutritional programs in the wider context of frailty and postoperative rehabilitation. A systematic review that included 6 studies and 665 patients with frailty reported that multicomponent exercise programs are associated with significantly improved global cognitive function [56].

## Preoperative Cognitive Training

Preoperative cognitive impairment is another potentially modifiable risk factor that has not yet been fully explored. Several studies have shown that cognitive training could improve baseline cognitive reserve. For example, a systematic review investigated the use of cognitive exercises in the critically ill patient population. Interventions studied included cognitive training exercises (such as memory and executive function), cognitive stimulation, and functional rehabilitation (development of new skills to cope with cognitive decline). The authors reported that the majority of included studies noted a drop in the incidence of delirium in association with the cognitive intervention programs [57]. A meta-analysis by Woods et al. [58] showed the effectiveness of cognitive stimulation in improving cognitive function, as well as quality of life and communication, for people with dementia. Rebok et al. [59] conducted a large-scale randomized controlled trial showing that cognitive training focused on memory, reasoning, and speed of processing improved cognitive function and performance of instrumental activities of daily living in community-dwelling older adults. A prospective cohort study by Tow et al. [60] found that older adults participating in cognitive activities, such as reading books, using email, singing, and playing computer games, had lower incidence and severity of delirium after undergoing elective orthopedic surgery. As a result, preoperative cognitive training may be a useful addition to multimodal surgical prehabilitation programs to reduce the risk of POD.

Rivera-Rivera et al. [61] showed that prehabilitation could catalyze cortical plastic changes in a study of five patients with WHO Grade II or III gliomas involving eloquent brain areas. While using continuous cortical electrical stimulation to suppress the functional areas within the tumor, patients would undergo a few weeks of behavioral training, such as different language tasks and motor functions. Intraoperative mapping and functional MRI showed new activation of brain areas that were previously inactive when performing the affected function, allowing for a more extensive tumor resection to improve prognosis while preserving cognitive function.

Kawano et al. [62] examined the effects of preoperative environmental enrichment on rats undergoing abdominal surgery. Preoperative environmental enrichment incorporated both physical activity with running wheels and cognitive activity with Hebb-Williams mazes. Cognitive function would then be assessed using a novel object recognition test, and hippocampal levels of proinflammatory cytokines would be measured. They found that aged rats in the sedentary group receiving surgery showed memory deficits and increased levels of TNF-α and IL-1β compared with young rats. This surgically induced cognitive impairment and neuroinflammation was attenuated in rats receiving preoperative environmental enrichment.

Humeidan et al. [63••] conducted the Neurobics Trial, a prospective randomized clinical trial, to determine whether cognitive prehabilitation could reduce the risk of POD in older patients undergoing major noncardiac, nonneurological surgery. A total of 268 patients were randomized preoperatively to receive electronic tablet-based brain exercise games or routine care. The brain exercises were completed on an electronic tablet-based software application, with various tasks focusing on memory, speed, attention, flexibility, and problem-solving. One hundred and twenty-five patients were randomized to the intervention arm and were instructed to complete 10 h of exercise before surgery. The authors reported that 28% of the patients completed between 0 and 2 h of exercise, while 9% of the patients completed the prescribed 10 h of exercise. Four patients were excluded from the intervention arm for not completing any exercises prior to surgery. Despite the relatively low number of patients who completed the recommended exercise duration, the authors reported that the intervention arm had significantly lower risk of delirium compared to the control arm when adjusted for frailty and surgical procedures (odds ratio 0.58, 95% confidence interval 0.33 to 0.99). While there was also a trend towards lower risk of delirium with longer duration of participation (control: 29%, less than 5 h: 18.4%, 5 to 10 h: 10.2%, more than 10 h: 9%), this was not statistically significant.

## Clinical Considerations

The Neurobics Trial demonstrated that even with suboptimal patient compliance (recommended duration of 10 h versus median participation of 4.6 h), preoperative cognitive exercises appear to reduce the risk of postoperative delirium [63••]. Vlisides et al. [64] conducted a randomized controlled trial in older adults prior to major surgery and concluded that home-based cognitive prehabilitation was not feasible. The intervention group was instructed to participate in cognitive training from their home computer for at least 7 days prior to their surgery that targeted executive function, attention, working memory, and visuospatial processing. However, only 17% of the intervention group were able to complete the 7 days of training. Reasons for declining enrollment or opting out of the study included feeling overwhelmed, time constraints, lack of computer access, and computer-related difficulties. Anxiety and time constraints may especially be significant issues in oncologic surgery due to the emotional stress associated with a cancer diagnosis and the limited time between diagnosis and surgical treatment. This limited time can be further taken up by numerous doctor appointments and other responsibilities. Despite most patients not completing the full 10 h of preoperative cognitive training in the Neurobics Trial, Humeidan et al. [63••] showed that preoperative cognitive training was indeed feasible with the intervention group having a significant reduction in postoperative delirium risk. They also found that those who had completed more than 5 h of preoperative cognitive training had around half the incidence of delirium as those who had completed less than 5 h. As a result, further research is needed to determine the optimal type, duration, and timing of preoperative cognitive training in order to balance efficacy while preserving patient compliance.

Surgical prehabilitation in oncology patients is further complicated by neoadjuvant therapies, such as chemotherapy and radiation. These therapies may contribute to further deconditioning and poor nutritional status from which patients may not have sufficient time for recovery before surgery, placing them at greater risk for postoperative complications [65]. This was seen in a study by West et al. [66] that showed that neoadjuvant chemoradiotherapy reduced physical fitness in patients with rectal cancer. However, a structured exercise program initiated after neoadjuvant chemoradiotherapy but before surgery could bring them back to baseline in just 6 weeks.

## Conclusion

Despite a greater understanding of the pathophysiology and risk factors associated with postoperative delirium, treatment options remain limited. Instead, the focus has been on risk stratification and reduction measures given the success of multicomponent interventions targeting these risk factors in hospitalized patients. These strategies are being adapted to the preoperative period with CGA and multimodal surgical prehabilitation. The promising results of the Neurobics Trial show that preoperative cognitive training could be a useful addition to this repertoire. However, further research still needs to be performed to determine the optimal type, duration, and timing. This is especially important in oncology patients whose frailty places them more at risk of surgical complications and may also be undergoing neoadjuvant therapies during the presurgical period.
